# Modified Buried Vertical Mattress Suture Combined With Tension‐Reducing Tape in Forearm Tattoo Resection—A Retrospective Study

**DOI:** 10.1111/jocd.70266

**Published:** 2025-05-29

**Authors:** Xuefeng Su, Xuchuan Zhou, Yueling Tang, Gejia Ma, Junzheng Wu, Bin Liu

**Affiliations:** ^1^ Department of Burn, Plastic and Cosmetic Surgery Xi'an Central Hospital, Xi'an Jiaotong University Xi'an China

**Keywords:** mechanical strain, modified buried vertical mattress suture, postoperative scar, tattoo, tension‐reducing tape

## Abstract

**Background:**

The Modified Buried Vertical Mattress Suture (MBVMS) has demonstrated potential in mitigating postoperative incision scar hyperplasia. This study aimed to retrospectively evaluate the efficacy of combining MBVMS with tension‐reducing tape in minimizing scar formation following forearm tattoo resection.

**Methods:**

A total of 46 patients undergoing forearm tattoo resection or partial resection were included. Participants were stratified into two cohorts: a control group (*n* = 17) receiving traditional intradermal sutures and a research group (*n* = 29) treated with MBVMS combined with tension‐reducing tape. Scar width was measured at predefined intervals (3, 6, and 12 months postoperatively). Scar hyperplasia was assessed using the Observer Scar Assessment Scale (OSAS) and Patient Scar Assessment Scale (PSAS).

**Results:**

Both groups exhibited initial scar widening between 3 and 6 months postoperatively, followed by gradual narrowing from 6 to 12 months. However, the research group demonstrated significantly reduced scar width at all time points compared to the control group (*p* < 0.05). Similarly, OSAS and PSAS scores were consistently lower in the research group (*p* < 0.05), indicating superior cosmetic and patient‐reported outcomes.

**Conclusion:**

These findings suggest that MBVMS combined with tension‐reducing tape effectively reduces scar hyperplasia in forearm tattoo resection, offering a promising approach for improving postoperative aesthetic outcomes.

## 1 Introduction

Tattoos are prevalent in approximately 10%–30% of the global population, with prevalence rates influenced by age, ethnicity, and demographic factors [1]. In China, an estimated 12.2% of individuals have tattoos, and growing cultural acceptance has led to increased interest in tattoos, paralleled by a rising demand for tattoo removal [2].

Current tattoo removal modalities—including chemical etching, laser ablation, dermabrasion, and surgical excision—are selected based on tattoo depth, size, location, and patient preference [3, 4, 5]. However, due to side effects such as incomplete removal, scarring, and pigmentation, none of these treatments have satisfactory cosmetic results.

It has been generally accepted that the applied suture technique is an important factor affecting wound healing and scar appearance [6]. The ideal suture technique requires reducing the mechanical tension of the wound, providing precise valgus and proximity to the edge of the wound, allowing the wound to be easily cared for, and not leaving traces on the skin from the suture material [7, 8].

Building on these principles, Shu et al. [9] introduced the Modified Buried Vertical Mattress Suture (MBVMS), which incorporates wedge‐shaped excision (WE) and heart‐shaped suture (HS) to mitigate postoperative scar hyperplasia. This technique diverges from traditional subcutaneous suturing by emphasizing tension redistribution and anatomical alignment [6, 9, 10].

This study retrospectively analyzed and evaluated the application of MBVMS combined with tension‐reducing tape in forearm tattoo resection, aiming to provide reference and guidance for reducing scar hyperplasia after tattoo resection.

## 2 Data and Methods

### 2.1 Inclusion and Exclusion Criteria

Inclusion criteria were as follows: (1) voluntary surgical treatment; (2) aged ≥ 18 years; and (3) had complete medical records (pre‐/postoperative photographs, detailed suture technique documentation, and tape compliance records) with a minimum of 12‐month follow‐up for scar assessment. Exclusion criteria were as follows: (1) predisposition to pathological scarring (personal/family history of keloids or prior abnormal scarring in the surgical area); (2) systemic conditions impairing wound healing (uncontrolled diabetes, scleroderma, chronic renal insufficiency, etc.); (3) postoperative use of corticosteroids or anti‐scarring agents (e.g., silicone gels); (4) concurrent scar‐modifying interventions (laser therapy and intralesional injections); and (5) incomplete follow‐up.

### 2.2 Patients

This retrospective cohort study enrolled 46 patients undergoing forearm tattoo resection at Xi'an Central Hospital (September 2018–December 2023), stratified into two groups: 17 controls receiving traditional intradermal sutures (pre‐December 2020; 7 males, 10 females) and 29 cases treated with modified buried vertical mattress suture (MBVMS) combined with tension‐reducing tape (post‐December 2020; 11 males, 18 females).

Baseline demographics and surgical variables were balanced between groups: mean age (control: 31.12 ± 8.41 years vs. research: 34.61 ± 9.19 years; *t* = 1.282, *p* = 0.207), incision length (control: 8.89 ± 3.85 cm vs. research: 11.34 ± 4.65 cm; *t* = 1.842, *p* = 0.072), and proportion of high‐tension zone incisions (control: 16.67% vs. research: 17.24%; *χ* ^2^ = 0.355, *p* = 0.551). High‐tension zones were defined as regions overlying dynamic structures (e.g., flexor tendon sheaths) or crossing wrist creases. All patients underwent preoperative screening for keloid predisposition (personal/family history, physical examination), with none meeting exclusion criteria. Gender distribution (*t* = 0.047, *p* = 0.828) showed no intergroup differences. This equipoise in baseline characteristics minimizes confounding and supports the comparative validity of the MBVMS technique's efficacy.

### 2.3 Operative Technique

#### 2.3.1 Incision Characteristics

Tattoo resection was performed along the peripheral margins of the tattoo design, typically adopting a spindle‐shaped excision pattern. For extensive tattoos requiring partial resection, this approach was determined through preoperative patient consultation to prioritize aesthetic and functional outcomes. In the research group, the surgical blade tilted inward to the center of the spindle and the skin at an angle of 60°, forming an oblique incision so that the cross‐section of the skin defect area after tattoo resection was trapezoidal.

#### 2.3.2 Suture Methods

In the control group, after the wound is fully stanched, it is better to remove the part of fat in the incision area according to the amount of fat between the deep and shallow fascia of each patient because the accumulation of fat after suture will increase the volume of the incision and increase the mechanical tension. According to the need, 2.0–5.0 cm was dissociated from the deep fascia layer to both sides of the incision. The wounds were sutured layer by layer with PDS‐II3‐0 and PDS‐II4‐0 sutures from deep to shallow. No tension reduction in the dermis characterizes the suture. Finally, the 6–0 nylon line was used to align the epidermis.

In the research group, the wound was fully stanched, and the adipose tissue near the incision was appropriately trimmed, and 2.0–4.0 cm was dissociated from the deep fascia layer to both sides of the incision as needed. The tension‐reducing suture between the deep fascia and the deep dermis was performed using the MBVMS method. From the deepfascia to the shallow fascia to the deep dermis, PDS‐II3‐0 suture was used. Technical method: (1) Deep fascia, superficial fascia, and deep dermis tissue: The first needle was inserted from the wound base to the direction of the incision and then rotated upward. The distance between the deep fascia and the superficial fascia was adjusted according to the thickness of the fat layer. The needle was removed from the dermis and superficial fascia 2–5 mm from the incision. Before the needle was removed, the suture was passed through the dermis. The length of the penetration was determined according to the actual thickness of the dermis, generally 2–5 mm. (2) The second needle was sutured to the opposite side of the wound edge. The method was carried out according to the principle of mirror image. The knot was tied to the deep surface of the superficial fascia so that the course of the suture was almost a “heart” shape. The whole wound skin accumulated to the middle of the incision, protruding the body surface by 3–6 mm; the appearance of the incision edge was slightly valgus, and the skin edge was well aligned. (3) PDS‐II5‐0 was used again on the skin 2–4 mm close to the edge of the incision, and the MBVMS method was repeated once to reduce the tension again, and the skin margin was tightly closed. (4) After the skin of the wound edge was aligned, the subcutaneous hemorrhage was squeezed out, the incision and its surrounding skin were wiped clean with normal saline, and the dry gauze was dried. First, a long, complete tension‐reducing tape was applied to the incision, and then short tape was applied longitudinally to maintain a tension‐free closure.

#### 2.3.3 Postoperative Incision Care

In general, the dressing was changed on the 2nd, 5th, and 7th day after the operation. The wound was scrubbed twice with a 1% iodophor cotton ball, washed with a normal saline cotton ball, dried with dry gauze, and bandaged with a sterile dressing. The sutures were removed 10 days after the operation in the control group. The patients in the research group were replaced with tension‐reducing tape once 2–5 days after the operation. Later, applying tension‐reducing tape was continued for 6–12 months. No patients in either group received postoperative laser therapy or topical anti‐scarring medications (e.g., silicone gels, corticosteroids, or onion extract preparations).

For patients undergoing staged resection of extensive tattoos, the secondary procedure may be scheduled as early as 6 months postoperatively, following confirmed stabilization of tissue remodeling. During the secondary resection, hypertrophic scar tissue from the initial surgery was excised concurrently, provided the patient exhibited adequate wound healing capacity and expressed a preference for further aesthetic refinement.

#### 2.3.4 Evaluation Indicators

Patients in both groups underwent standardized follow‐up assessments at 6 and 12 months postoperatively. Scar evaluation was conducted using the validated Patient and Observer Scar Assessment Scale (POSAS) 2.0, which comprises two distinct components: the Observer Scar Assessment Scale (OSAS) and the Patient Scar Assessment Scale (PSAS). For both scales, each of the six items is independently scored on a 10‐point Likert scale (1 = closest to normal skin, 10 = worst possible symptom/feature), and the total score is calculated as the sum of all six item scores. The PSAS evaluates patient‐reported symptoms, including pain, itching, color mismatch, stiffness, perceived thickness, and irregularity, while the OSAS assesses clinician‐graded parameters: vascularity, pigmentation, objective thickness, surface relief, pliability, and surface area. Thus, the total score for each scale (OSAS and PSAS) ranges from 6 to 60 points, with higher scores indicating poorer scar quality. In this study, both total scores and individual item scores were recorded to allow comprehensive analysis; however, primary outcome comparisons focused on the aggregated total scores to reflect overall scar severity, consistent with POSAS validation protocols [6, 11, 12, 13]. To address limitations of the POSAS in quantifying scar width, the maximal scar width was measured using digital calipers (Mitutoyo, Kawasaki, Japan) under standardized lighting. Postoperative complications, including wound dehiscence, hematoma, infection, and suture reactivity, were documented.

### 2.4 Statistical Analyses

SPSS 22.0 statistical software was used to analyze the data. The measurement data conforming to the normal distribution were expressed as mean ± standard deviation ($\bar{x}$ ± s). The independent sample *t*‐test, paired sample *t*‐test, and two‐factor repeated measurement analysis of variance were used to compare groups. Those that did not conform to the normal distribution were expressed by median and quartile, and the Mann–Whitney *U* test was used to compare groups. The count data were expressed as a rate (%) and analyzed by chi‐square (*χ* ^2^) test. *p* < 0.05 indicated that the difference was statistically significant.

## 3 Results

In the control group, 7 patients (41.18%) underwent complete tattoo resection, while 10 (58.82%) underwent partial resection due to the large tattoo size. In the research group, 14 patients (48.28%) underwent complete resection, and 15 (51.72%) underwent partial resection for the same reason. No infections or suture rejection occurred during follow‐up. In the control group, one patient developed an 8 mm incision dehiscence 10 days postoperatively due to strenuous activity, which healed after secondary suturing following debridement. Another patient developed a tension blister at the incision margin, which resolved after puncturing and dressing changes. In the research group, three patients experienced mild dermal pigmentation near the incision edge.

### 3.1 Comparison of OSAS Scores at 3, 6, and 12 Months After Operation

**FIGURE 1:**
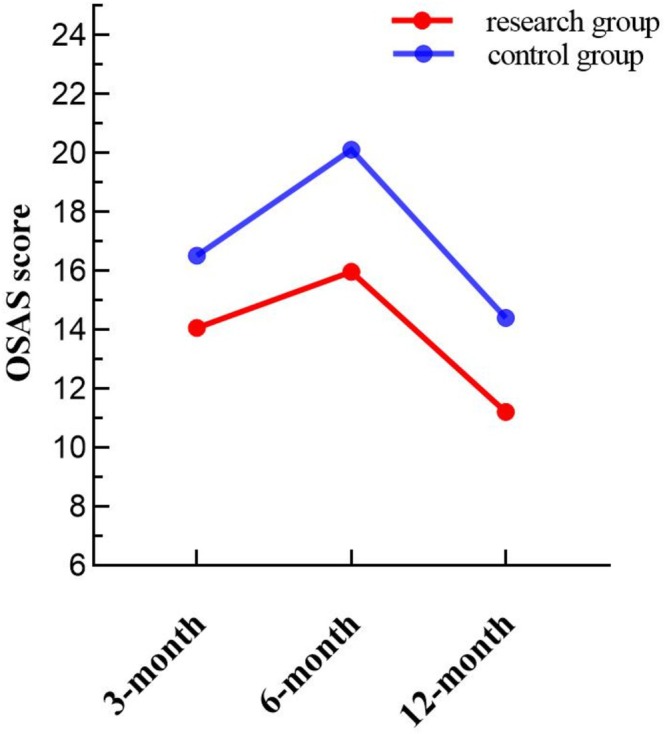
Comparison of OSAS scores between the two groups at different time points.

The OSAS scores at different time points are detailed in Table 1. Mauchly's test indicated a violation of the sphericity assumption (*p* = 0.001 < 0.05). Given an epsilon (*ε*) of 0.671 (< 0.75), the Greenhouse–Geisser correction was applied for repeated measures ANOVA. The analysis revealed a significant main effect of intervention (*F* = 8.367, *p* = 0.006) and a significant interaction between intervention and time (*F* = 3.875, *p* = 0.042). Differences across time points were also statistically significant (*F* = 147.694, *p* < 0.001). As shown in Figure 1, OSAS scores increased from 3 to 6 months postoperatively, with a smaller increase in the research group. From 6 to 12 months, scores declined, with a smaller decrease observed in the research group compared to the control group.

**TABLE 1:** Comparison of OSAS scores, PSAS scores and scar width (mm) between the two groups at different times.

		3‐month follow‐up	6‐month follow‐up	12‐month follow‐up	*F*	*p*
OSAS score	Control group	16.53 ± 4.00	20.12 ± 4.50	14.41 ± 3.87	147.694	0.001
	Research group	14.07 ± 3.68	15.97 ± 3.91	11.21 ± 3.57		
PSAS score	Control group	16.94 ± 3.86	20.71 ± 4.03	15.35 ± 3.57	101.981	0.001
	Research group	14.45 ± 4.02	16.38 ± 3.99	12.24 ± 3.09		
Scar width	Control group	2.52 ± 0.54	2.82 ± 0.51	2.49 ± 0.40	9.238	0.001
	Research group	1.56 ± 0.37	1.72 ± 0.32	1.31 ± 0.37		

Abbreviations: OSAS, Observer Scar Assessment Scale; PSAS, Patient Scar Assessment Scale.

### 3.2 Comparison of PSAS Scores at 3, 6, and 12 Months After Operation

**FIGURE 2:**
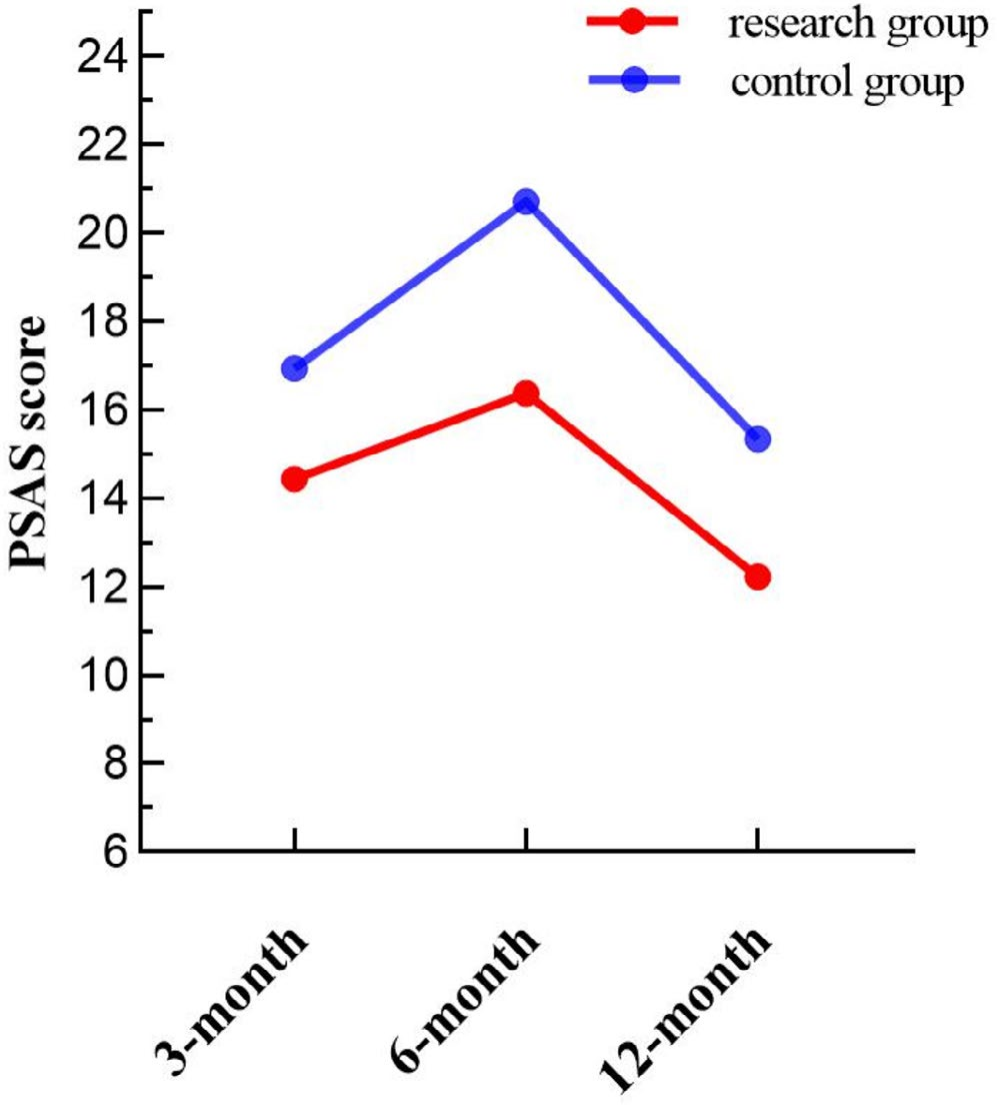
Comparison of PSAS scores between the two groups at different time points.

The PSAS scores at different time points are detailed in Table 1. Mauchly's test confirmed that the data met the sphericity assumption (*p* = 0.053 > 0.05). Repeated measures ANOVA revealed a significant main effect of intervention (*F* = 9.358, *p* = 0.004) and a significant interaction between intervention and time (*F* = 3.889, *p* = 0.024). Differences across time points were also statistically significant (*F* = 101.981, *p* < 0.001). As shown in Figure 2, the PSAS scores increased from 3 to 6 months postoperatively, with a smaller increase observed in the research group compared to the control group. From 6 to 12 months, scores declined, with the research group experiencing a smaller decrease.

### 3.3 Comparison of Scar Width at 3, 6, and 12 Months After Operation

**FIGURE 3:**
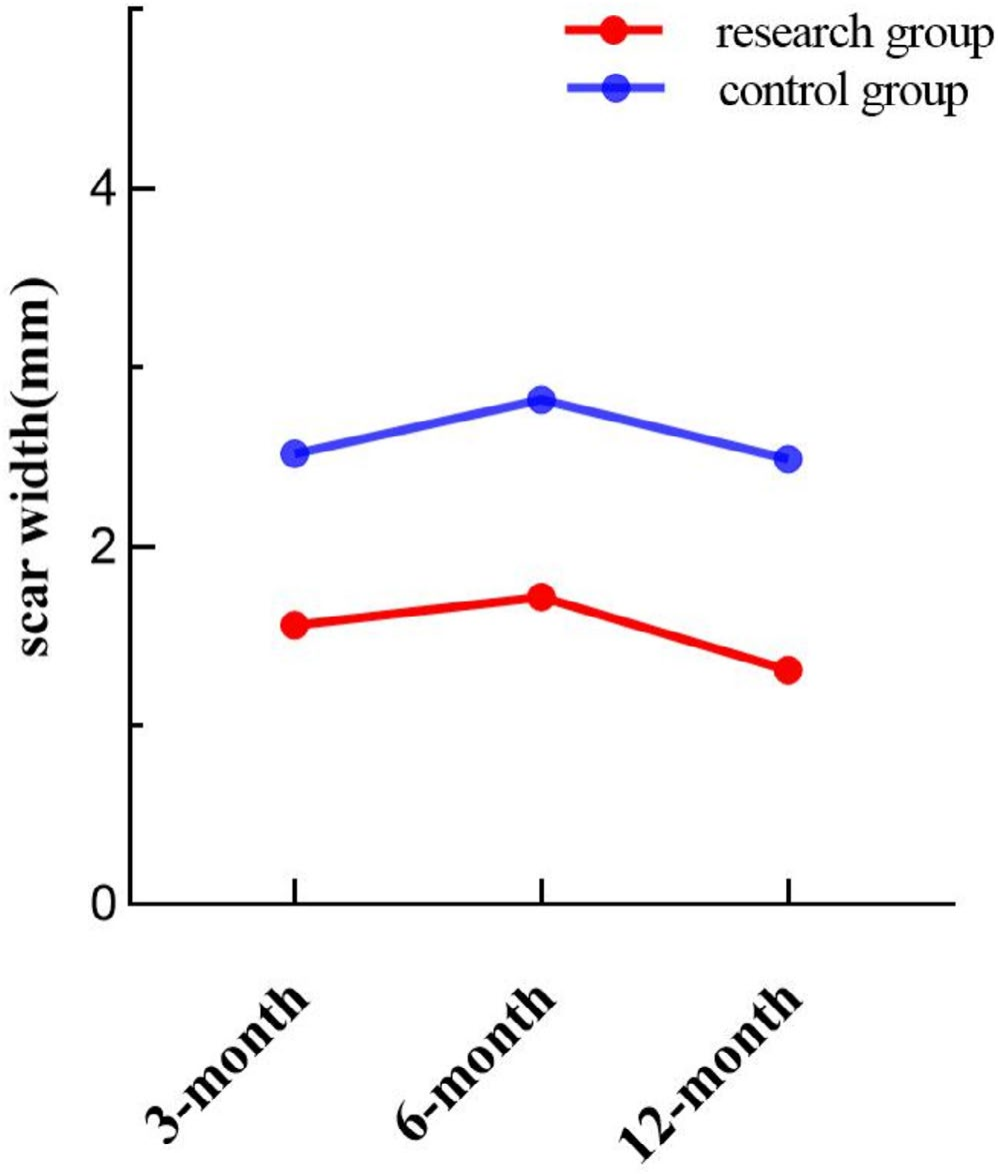
Comparison of scar width between the two groups at different time points.

The scar width at different time points is detailed in Table 1. Mauchly's test confirmed that the data met the sphericity assumption (*p* = 0.582 > 0.05). Repeated measures ANOVA showed a significant main effect of intervention (*F* = 213.789, *p* < 0.001) but no significant interaction between intervention and time (*F* = 0.869, *p* = 0.423). Differences between time points were statistically significant (*F* = 9.238, *p* < 0.001). As shown in Figure 3, the scar widened from 3 to 6 months postoperatively, with a smaller increase in the research group. From 6 to 12 months, the scar gradually narrowed, with a greater reduction observed in the research group compared to the control group.

### 3.4 Typical Case

**FIGURE 4:**
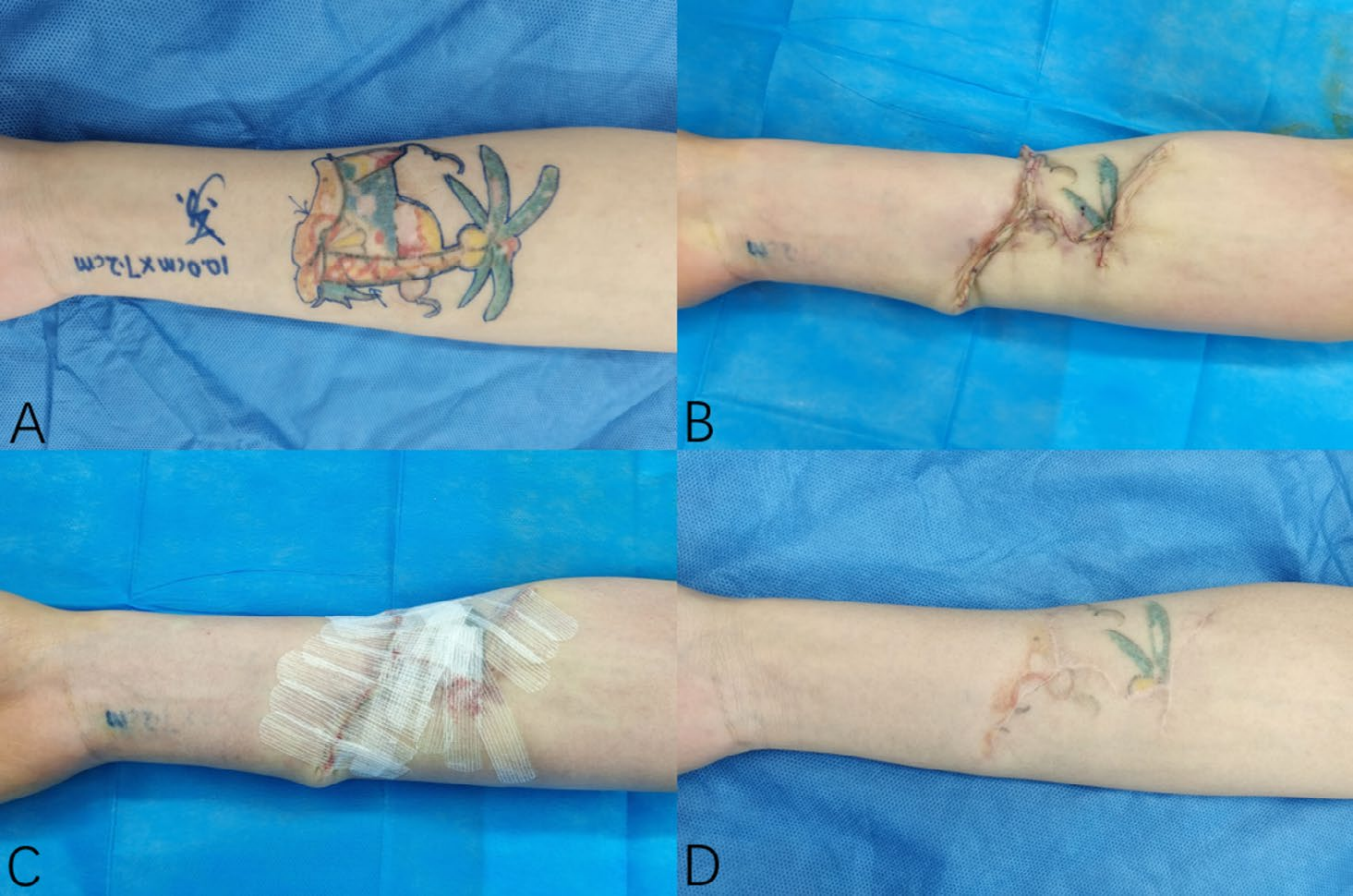
(A) Before the operation; (B) immediate postoperative; (C) sticking tension‐reducing tape to the wound; (D) 12‐month follow‐up.

Case A: A 27‐year‐old female complained of tattooing on her inside right forearm for 4 years. Physical examination: An irregular color tattoo about 10.0 cm × 7.2 cm in size can be seen on the right forearm, and scar hyperplasia can be seen on the surface after laser treatment, without skin ulceration. Diagnosis: Right forearm tattoo. The preoperative examination was completed, the part of right forearm tattoo resection was performed, and the wound was closed by MBVMS combined with tension‐reducing tape. The tension‐reducing tape was replaced once 2–5 days after operation, and the tension‐reducing tape was continuously applied for 6–12 months later. The scar width was 1.4, 1.6, and 1.3 mm at 3, 6, and 12 months after operation, respectively. OSAS scores were 13, 15, and 12, respectively. PSAS scores were 14, 16, and 13, respectively. The patients were satisfied with the treatment effect. (Figure 4).

**FIGURE 5:**
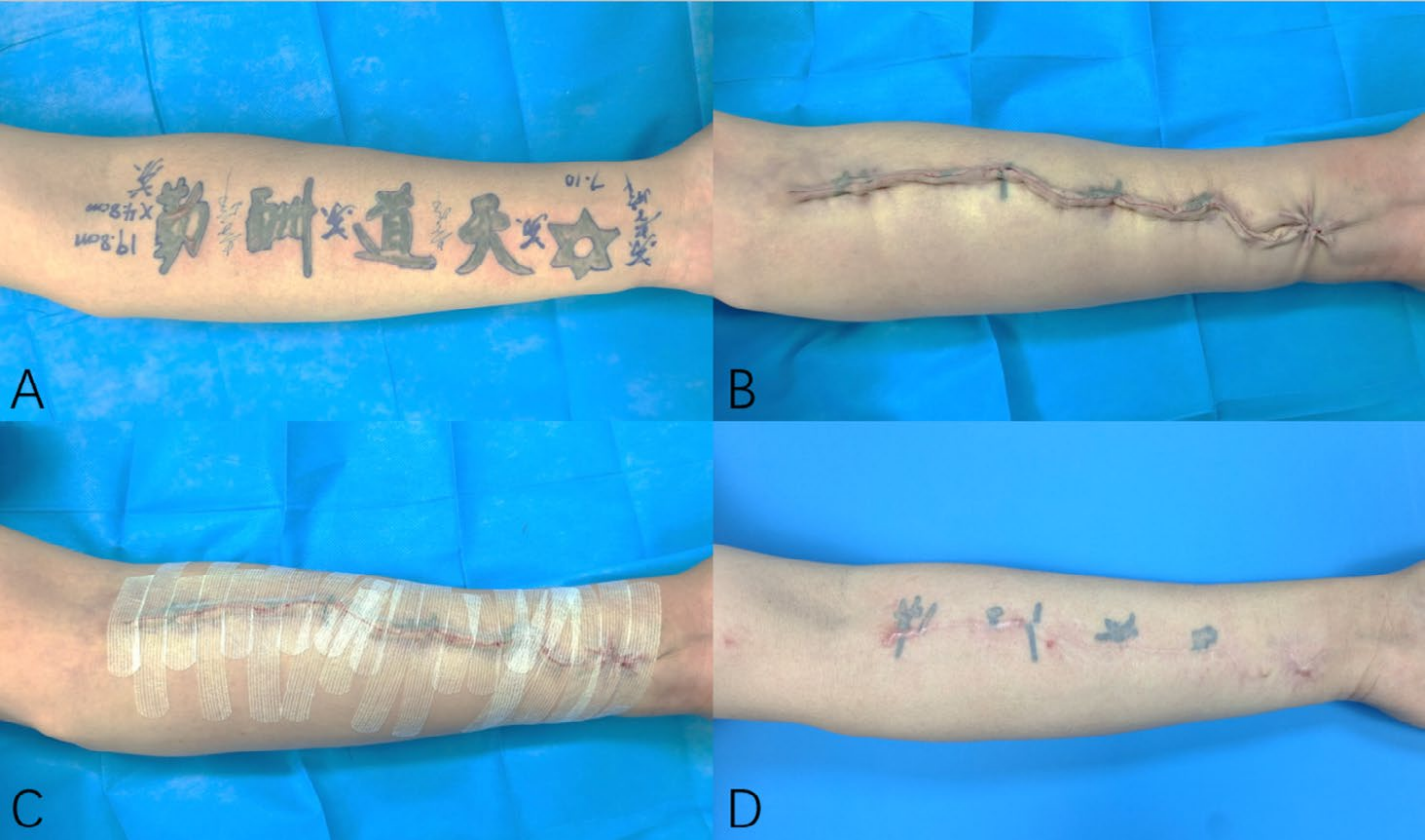
(A) Before the operation; (B) immediate postoperative; (C) sticking tension‐reducing tape to the wound; (D) 12‐month follow‐up.

Case B: A 32‐year‐old male complained of tattooing on his inside left forearm for 6 years. Physical examination: An irregular black tattoo about 19.8 cm × 4.8 cm in size can be seen on the left forearm, without skin ulceration. Diagnosis: Left forearm tattoo. The preoperative examination was completed, the part of left forearm tattoo resection was performed, and the wound was closed by MBVMS combined with tension‐reducing tape. The tension‐reducing tape was replaced once 2–5 days after operation, and the tension‐reducing tape was continuously applied for 6–12 months later. The scar width was 1.9, 1.9, and 1.7 mm at 3, 6, and 12 months after operation, respectively. OSAS scores were 16, 19, and 13, respectively. PSAS scores were 15, 17, and 15, respectively. The patients were satisfied with the treatment effect (Figure 5).

## 4 Discussion

The tattoo art was recorded in human civilization thousands of years ago [14]. Different from Western countries, China has used tattoos as a penalty since ancient times and continued to the Ming and Qing Dynasties, so the recognition of tattoos is low. Propelled by sustained socioeconomic advancement and evolving sociocultural perceptions in contemporary China, the population of tattoos is on the rise, and the demand for tattoos is growing. The tattoo dye market needs more supervision. In China, some tattoos are carried out by unlicensed practitioners in places where hygiene does not meet the requirements. The quality of the dyes is uneven, resulting in a variety of complications for patients. Considering the varying professional requirements for tattoos and changes in personal aesthetic preferences, some individuals seek to remove such permanent markings.

Lasers have largely supplanted historical tattoo removal techniques such as chemical etching and dermabrasion due to their capacity for selective pigment targeting, which minimizes collateral damage to surrounding tissues. However, the efficacy of laser therapy is inherently limited by the diverse absorption spectra of tattoo pigments, restricting the availability of optimal laser wavelengths and complicating outcome predictability. To mitigate this variability, preliminary testing on small, localized areas is strongly advised to assess treatment response, with black and red pigments demonstrating the highest clearance rates. Despite these precautions, patient dissatisfaction may arise due to the inconsistent results observed with colored tattoos: while some pigments are fully eradicated, others exhibit partial fading or no response.

Furthermore, laser removal may induce unintended cutaneous changes, including scar formation and dyspigmentation (hyper‐ or hypopigmentation), particularly as underlying skin alterations become apparent post‐tattoo clearance. Consequently, some patients with small‐area tattoos opt for direct surgical resection to achieve complete removal, though concerns regarding postoperative scar hyperplasia persist. This underscores the critical need for strategies to attenuate postsurgical scar formation, a challenge addressed in this study through the application of the MBVMS combined with tension‐reducing techniques.

Many factors contribute to the formation of scars, such as age, systemic nutritional status, wound inflammation, foreign body retention, and local blood circulation [15, 16, 17, 18]. Mechanical tension plays an important role in the formation of scars during wound healing [19]. The mechanical tension acting on the wound is composed of dynamic force (external force composed of potential muscle fibers) and static force (internal force of the dermal component of the skin around the wound) [20]. The basic elements of postoperative incision scar control are good wound margin, mild valgus, and effective tension reduction. Effective tension reduction can avoid the formation of depression, and the tension of the wound edge not only affects the width of the scar but also is an important stimulating factor for scar hyperplasia [21, 22].

We used MBVMS to perform the first tension reduction step by step between the deep fascia, shallow fascia, and dermis; the second tension reduction was performed between the surface of the superficial fascia and the dermis. The two tension reductions solved the problem of tension in dynamic force and static force so that the incision was well stacked and valgus, and the parallel lines of 0.5–1.0 cm next to the incision formed a dermal “orange peel‐like” change, forming a state of full tension reduction.

This dual‐phase tension‐reduction strategy offers distinct advantages over conventional tension‐relieving sutures (e.g., simple interrupted or traditional vertical mattress techniques). First, the layered, stepwise redistribution of tension across fascial planes minimizes focal stress points, which are common in single‐layer closure methods and predispose to scar widening under dynamic movement. Second, the buried vertical mattress design creates a broader dermal “cushion” that disperses tension radially, rather than concentrating it linearly along the incision—a critical feature for mobile regions like the forearm. Third, the synergistic use of tension‐reducing tape provides prolonged, uniform off‐loading during the proliferative phase of healing, complementing the suture's mechanical stability. In contrast, traditional methods relying solely on sutures often exhibit gradual tension loss due to suture creep or early absorption, while tape‐alone approaches lack deep fascial anchorage. Biomechanically, this hybrid technique achieves a balance between immediate intraoperative tension relief and sustained postoperative support, reducing the risk of hypertrophic scarring caused by cyclical mechanical stress.

Following placement of MBVMS with PDS‐II3‐0 long‐term absorbable sutures (Ethicon, Somerville, NJ), the elevated wound edges typically resolve into a flattened contour within approximately 3–5 months. This timeline correlates with the hydrolysis profile of PDS‐II, which retains approximately 50% tensile strength at 6 weeks and undergoes complete absorption by 180 days, providing sustained mechanical support during early remodeling [23]. In high‐tension anatomical zones, earlier flattening (2–4 months) may occur due to enhanced tension offloading from the combined suture‐tape system, which reduces repetitive shear forces and accelerates tissue alignment. Preoperative counseling emphasized this transient elevation as an expected physiological response, with persistent raised morphology beyond 6 months warranting evaluation for hypertrophic scarring or delayed suture reactivity.

The prevention and treatment of scars should run through the early stage after trauma, immature scar, and scar shape [16, 24]. The healing after the incision suture is a process of gradual recovery of tension. The early scar has weak tensile strength and gradually recovers to 80% of normal skin within 1 year after surgery. The mechanical tension of the edge runs through all stages, so local tension reduction should run through the whole wound treatment process [25]. Local tension reduction should last at least 3 months in clinical application, and it should last for 6 months. Tape tension reduction is often used after trauma surgery because of its effective and convenient characteristics. Tape tension reduction can reduce the mechanical tension of the wound and can also reduce hypertrophic scars by imitating the stratum corneum and promoting healing. Therefore, to have a good continuous tension reduction effect, we use the method of applying suture‐free adhesive tape outside the skin to reduce the effect of static force to achieve continuous tension reduction. In use, we observed that the tension‐reducing tape not only has a tension‐reducing effect in the horizontal direction but also tends to inhibit the outward growth of scars in the vertical direction of the skin, thus forming a region above the space to inhibit the outward growth of scars.

In this study, the tension of incision healing was reduced by using the method of super tension suture of the dermis and deep fascia layer combined with external tension tape of incision to achieve tension‐free healing and minimize incision scar. According to the POSAS score and scar width measurement results of the treatment group and the control group, the effect of the study group was significantly better than the traditional tension‐reducing suture method of the control group. In the process of treating hypertrophic scars, the key point is to use the improved buried vertical mattress suture to reduce the tension of the skin and subcutaneous tissue two times, thus forming a super tension reduction state. Absorbable sutures with long tension were selected to bear the tension of the skin and subcutaneous tissue near the incision. After the operation, the tension‐reducing tape was applied for 6 months.

Based on retrospective outcomes and biomechanical analyses, this combined technique demonstrates efficacy for both monochromatic (e.g., black/gray) and multicolored tattoos, though wider excision margins may be required for densely pigmented designs to ensure complete ink removal, necessitating meticulous tension management during closure. For extensive tattoos, staged resection at 6‐month intervals is recommended to mitigate excessive wound tension, with the secondary procedure optimally timed during the tissue remodeling phase (5–7 months postoperatively) to leverage stabilized scar maturation. Priority should be given to tattoos located in low‐tension zones (e.g., proximal forearm), where reduced dynamic shear forces enhance suture‐tape synergy, whereas high‐mobility regions (e.g., volar wrist) warrant cautious patient selection due to elevated mechanical stress. Younger patients (18–40 years) with Fitzpatrick skin types I–III exhibit superior outcomes owing to favorable dermal elasticity, while older individuals (> 60 years) may require preoperative skin laxity assessments.

This study has several limitations. First, the sample size was small, which may restrict the statistical power of the findings. Second, the representativeness of the cohort was limited, as the study focused exclusively on forearm tattoo resection scars and did not include high‐tension anatomical regions known to be prone to scarring, such as the upper arm or sternum. Third, the methodological design was constrained by its single‐center framework, involvement of a single surgical site (forearm), and reliance on one surgeon, all of which may reduce the external validity of the results. Future multicenter studies incorporating diverse anatomical regions and multiple surgeons are warranted to validate these findings and enhance generalizability.

## 5 Conclusion

This study provides preliminary evidence that the Modified Buried Vertical Mattress Suture (MBVMS) combined with tension‐reducing tape effectively reduces scar hyperplasia following forearm tattoo resection, which was worthy of further clinical promotion.

## Author Contributions

Xuefeng Su, Xuchuan Zhou, Yueling Tang, Gejia Ma, Junzheng Wu, and Bin Liu designed and conducted this study, including patient recruitment, data collection, and data analysis. Xuefeng Su interpreted the data and drafted the manuscript. Xuchuan Zhou mainly designed the study. Yueling Tang interviewed the participants. Gejia Ma and Junzheng Wu collected and analyzed the data. Bin Liu was the leader of this study; he provided technical material support and reviewed the write‐up and submission. All authors have read and approved the final manuscript.

## Conflicts of Interest

The authors declare no conflicts of interest.

## Data Availability

The data that support the findings of this study are available from the corresponding author upon reasonable request.

## References

[1] N. Kluger , “An Update on Cutaneous Complications of Permanent Tattooing,” Expert Review of Clinical Immunology 15, no. 11 (2019): 1135–1143, 10.1080/1744666X.2020.1676732. 31594417

[2] N. Kluger , S. Seité , and C. Taieb , “The Prevalence of Tattooing and Motivations in Five Major Countries Over the World,” Journal of the European Academy of Dermatology and Venereology 33, no. 12 (2019): e484–e486, 10.1111/jdv.15808. 31310367

[3] L. Hernandez , N. Mohsin , F. S. Frech , I. Dreyfuss , A. Vander Does , and K. Nouri , “Laser Tattoo Removal: Laser Principles and an Updated Guide for Clinicians,” Lasers in Medical Science 37, no. 6 (2022): 2581–2587, 10.1007/s10103-022-03576-2. 35604505

[4] G. Dash , A. Patil , M. Kassir , et al., “Non‐Laser Treatment for Tattoo Removal,” Journal of Cosmetic Dermatology 22, no. 1 (2023): 74–78, 10.1111/jocd.14819. 35122391

[5] P. Hirtler and J. Serup , “A Practical Approach to Cosmetic Tattoo Removal With the Nd:YAG Laser,” Current Problems in Dermatology 56 (2022): 259–267, 10.1159/000521483. 37263203

[6] Z. Liu , Z. Tang , X. Hao , et al., “Modified Buried Vertical Mattress Suture Versus Buried Intradermal Suture: A Prospective Split‐Scar Research,” Dermatologic Surgery 47, no. 3 (2021): e75–e80, 10.1097/DSS.0000000000002642. 32796329

[7] C. G. Regula and C. Yag‐Howard , “Suture Products and Techniques: What to Use, Where, and Why,” Dermatologic Surgery 41 (2015): S187–S200, 10.1097/DSS.0000000000000492. 26418685

[8] C. Yag‐Howard , “Sutures, Needles, and Tissue Adhesives,” Dermatologic Surgery 40 (2014): S3–S15, 10.1097/01.DSS.0000452738.23278.2d. 25158874

[9] X. Zhang , J. S. Diao , S. Z. Guo , et al., “Wedge‐Shaped Excision and Modified Vertical Mattress Suture Fully Buried in a Multilayered and Tensioned Wound Closure,” Aesthetic Plastic Surgery 33, no. 3 (2009): 457–460, 10.1007/s00266-009-9311-6. 19387723

[10] Z. Liu , X. Liu , L. He , et al., “Different Suturing Techniques in Thoracic Incision: Protocol for a Feasibility Randomised Controlled Trial,” BMJ Open 9, no. 1 (2019): e021645, 10.1136/bmjopen-2018-021645. PMC6340629 30782673

[11] W. Ting , Y. Chong , J. Xu , J. Huang , N. Yu , and Z. Liu , “Treatment of Keloids Using Plasma Skin Regeneration Combined With Radiation Therapy Under the Evaluation of Patient and Observer Scar Assessment Scale,” Clinical, Cosmetic and Investigational Dermatology 14 (2021): 981–989, 10.2147/CCID.S321348. 34385829 PMC8353170

[12] L. Lin , P. Guo , X. Wang , et al., “Effective Treatment for Hypertrophic Scar With Dual‐Wave‐Length PDL and Nd:YAG in Chinese Patients,” Journal of Cosmetic and Laser Therapy 21, no. 4 (2019): 228–233, 10.1080/14764172.2018.1516889. 30260709

[13] J. H. Chung , S. H. Kwon , K. J. Kim , et al., “Reliability of the Patient and Observer Scar Assessment Scale in Evaluating Linear Scars After Thyroidectomy,” Advances in Skin & Wound Care 34, no. 6 (2021): 1–6, 10.1097/01.ASW.0000744344.46898.6e. 33979825

[14] I. Kurniadi , F. Tabri , A. Madjid , A. I. Anwar , and W. Widita , “Laser Tattoo Removal: Fundamental Principles and Practical Approach,” Dermatologic Therapy 34, no. 1 (2021): e14418, 10.1111/dth.14418. 33068020

[15] Z. C. Wang , W. Y. Zhao , Y. Cao , et al., “The Roles of Inflammation in Keloid and Hypertrophic Scars,” Frontiers in Immunology 11 (2020): 603187, 10.3389/fimmu.2020.603187. 33343575 PMC7746641

[16] H. J. Lee and Y. J. Jang , “Recent Understandings of Biology, Prophylaxis and Treatment Strategies for Hypertrophic Scars and Keloids,” International Journal of Molecular Sciences 19, no. 3 (2018): 711, 10.3390/ijms19030711. 29498630 PMC5877572

[17] A. Knowles and D. A. Glass, 2nd , “Keloids and Hypertrophic Scars,” Dermatologic Clinics 41, no. 3 (2023): 509–517, 10.1016/j.det.2023.02.010. 37236718

[18] L. Liu , Y. Xue , Y. Chen , et al., “Prevalence and Risk Factors of Acne Scars in Patients With Acne Vulgaris,” Skin Research and Technology 29, no. 6 (2023): e13386, 10.1111/srt.13386. 37357642 PMC10240192

[19] B. Kuehlmann , C. A. Bonham , I. Zucal , L. Prantl , and G. C. Gurtner , “Mechanotransduction in Wound Healing and Fibrosis,” Journal of Clinical Medicine 9, no. 5 (2020): 1423, 10.3390/jcm9051423. 32403382 PMC7290354

[20] H. I. Harn , R. Ogawa , C. K. Hsu , M. W. Hughes , M. J. Tang , and C. M. Chuong , “The Tension Biology of Wound Healing,” Experimental Dermatology 28, no. 4 (2019): 464–471, 10.1111/exd.13460. 29105155

[21] T. Dohi , J. Padmanabhan , S. Akaishi , et al., “The Interplay of Mechanical Stress, Strain, and Stiffness at the Keloid Periphery Correlates With Increased Caveolin‐1/ROCK Signaling and Scar Progression,” Plastic and Reconstructive Surgery 144, no. 1 (2019): 58e–67e, 10.1097/PRS.0000000000005718. 31246819

[22] C. K. Hsu , H. H. Lin , H. I. Harn , et al., “Caveolin‐1 Controls Hyperresponsiveness to Mechanical Stimuli and Fibroblasts,” Journal of Investigative Dermatology 138, no. 1 (2018): 208–218, 10.1016/j.jid.2017.05.041. 28899682

[23] Ethicon , “PDS II (Polydioxanone) Sutures: Product Information,” (2021).

[24] R. Ogawa , T. Dohi , M. Tosa , M. Aoki , and S. Akaishi , “The Latest Strategy for Keloid and Hypertrophic Scar Prevention and Treatment: The Nippon Medical School (NMS) Protocol,” Journal of Nippon Medical School 88, no. 1 (2021): 2–9, 10.1272/jnms.JNMS.2021_88-106. 32741903

[25] S. Fu , A. Panayi , J. Fan , et al., “Mechanotransduction in Wound Healing: From the Cellular and Molecular Level to the Clinic,” Advances in Skin & Wound Care 34, no. 2 (2021): 67–74, 10.1097/01.ASW.0000725220.92976.a7. 33443911

